# COVID-19 pandemic and psychological fatigue in Turkey

**DOI:** 10.1177/0020764020941889

**Published:** 2020-07-10

**Authors:** Ebru Morgul, Abdulbari Bener, Muhammed Atak, Salih Akyel, Selman Aktaş, Dinesh Bhugra, Antonio Ventriglio, Timothy R Jordan

**Affiliations:** 1 Department of Psychology, School of Humanities and Society Sciences, Ibn Haldun University, Istanbul, Turkey; 2 Department of Biostatistics & Medical Informatics, Cerrahpaşa Faculty of Medicine, Istanbul University, Istanbul, Turkey; 3 Department of Evidence for Population Health Unit, School of Epidemiology and Health Sciences, The University of Manchester, Manchester, UK; 4 Department of Public Health, Medipol International School of Medicine, Istanbul Medipol University, Istanbul, Turkey; 5 Department of Biostatistics & Medical Informatics, Cerrahpaşa Faculty of Medicine, Istanbul University Cerrahpaşa, and International School of Medicine, Istanbul Medipol University, Istanbul, Turkey; 6 Department of Public Health, Çapa Faculty of Medicine, Istanbul University, Istanbul, Turkey; 7 Department of Psychiatry, HSPRD, Institute of Psychiatry, King’s College London, London, UK; 8 Department of Clinical & Experimental Medicine, University of Foggia, Foggia, Italy

**Keywords:** COVID-19, fatigue, pandemic, infectious disease, public health, perceptions, KAP study

## Abstract

**Aim:**

The aim of this study was to investigate the association between the COVID-19 pandemic and psychological fatigue as a mental health issue among the population of Istanbul, Turkey.

**Participants and methods:**

This is a cross-sectional study conducted in Istanbul, Turkey, between March and June 2020, where a total of 4,700 persons were approached and 3,672 (78%) of participants (64.4% males and 35.6% females) completed the Knowledge Attitude Practices (KAP) and Fatigue Assessment Scale (FAS) questionnaires.

**Results:**

In this study, 64.1% of participants were categorized as psychologically fatigued and 35.9% as normal. There was a significant difference between fatigued and normal participants with respect to age, educational level, occupational status, place of residence and number of family members (*p* < .001). Other differences related to knowledge of COVID-19 were symptoms, treatment, ways of spreading (*p* < .001), prevention by avoiding crowded places (*p* = .008) and isolation (*p* = .002). For attitudinal items, normal participants generally showed more positive attitudes than the fatigued in believing that COVID-19 will finally be controlled, satisfaction with preventive measures taken by the authorities, reporting suspected cases with symptoms and trusting that Turkey can overcome the COVID-19 pandemic (*p* < .001). Multivariate stepwise regression analysis indicated that level of education, avoiding going to crowded places, eye, nose and mouth organs are sensitive organs to the virus, keeping physical distance due to epidemic affect by COVID-19 virus, isolation and treatment of people reduce the spread of COVID-19 virus and 14-days period of time, COVID-19 is mainly transmitted through contact with the respiratory droplets of an infected person, occupational status, health education programme needed and antibody treatment variables were significantly associated with fatigue after adjusting for age, gender and income variables.

**Conclusion:**

The current study provides valuable information for policymakers and mental health professionals worldwide regarding associations between the mental health of individuals and the ongoing outbreak, COVİD-19.

## Introduction

In mid-December 2019, the Chinese city of Wuhan reported a novel pneumonia caused by a corona virus disease which has spread domestically and internationally since then (Huang et al., 2020; WHO, 2020b). The virus has been named specifically as severe acute respiratory syndrome coronavirus 2 (SARS-CoV-2; Huang et al., 2020; WHO, 2020b; Sohrabi et al., 2020), but is known more generally as COVID-19, and is highly infectious. Its main clinical symptoms include fever, fatigue or myalgia, dry cough and shortness of breath or difficulty breathing (Nicola et al., 2020; Tian et al., 2020; Wang et al., 2020) and the World Health Organization (WHO, 2020a) declared the COVID-19 outbreak a public health emergency of international concern and a pandemic.

The COVID-19 pandemic has resulted in day-to-day significant existential stress associated with the loss of many patients, colleagues or loved ones (WHO, 2020b). In addition to health-related problems, the pandemic has also created financial problems and fears of an imminent economic crisis and depression. Due to the pandemic, many workplaces, including factories, schools and universities, have been closed down. Preventive measures such as self-isolation, travel restrictions and lockdown forced a decrease in the workforce across all economic sectors, and the need for medical products has significantly increased (Nicola et al., 2020; Tian et al., 2020). In the face of such a global pandemic many people might have been faced with the fear of health and safety for themselves, their families and significant others, as well as fear for their jobs or finances. COVID-19 not only represents the appearance of a new virus but it has also become an economic burden and a major psycho-social problem. Undoubtedly, COVID-19 poses a challenge to psychological resilience.

Psychological factors play a vital role during any pandemic. A population’s attitudes towards vaccination and social distancing, for example, have critical effects on the spread of the infection. In addition, psychological factors are related to how people cope with the threat of infection, fear of losing loved ones and the grief of actually losing loved ones may cause increased levels of psychological distress. Those with pre-existing psychological conditions, such as anxiety disorders, may have these conditions worsened (Taylor, 2019). In a cross-sectional study, Wang et al. (2020) assessed levels of depression, stress and anxiety at the beginning of the COVID-19 outbreak, and found that 53.8% of participants showed the evidence of severe psychological impacts of the outbreak. The ongoing COVID-19 epidemic is inducing even more fear, and an understanding of its effect on mental health status is urgently needed for society (Finsterer & Mahjoub, 2014; Lai et al., 2020; Lewis & Wessely, 1992; Matias et al., 2020; Taylor, 2019; Yao et al., 2020; Zhong et al., 2020).

Fatigue, one of the listed symptoms of COVID-19 (Huang et al., 2020; Nicola et al., 2020; Sohrabi et al., 2020; Tian et al., 2020; Wang et al., 2020; WHO, 2020b), has a special characteristic in that it can be a symptom of a physical disease, as well as a manifestation of an underlying psychological issue, or both. Despite being a commonly observed complaint among the general population, fatigue is considered as a prevalent symptom of various physical and psychiatric disorders (Lewis & Wessely, 1992). In their review (Finsterer & Mahjoub, 2014), authors have listed various conditions that influence the experience of fatigue, including age, gender, physical condition, type of food, mental status, psychological conditions, personality and health status. Indeed, fatigue can be a condition occurring under stress, exercise or rest, a physiological reaction, pathological reaction or a mere symptom of a disease (Lai et al., 2020; Matias et al., 2020;Yao et al., 2020). In addition to the health-related and financially distressing conditions of the COVID-19 pandemic, self-isolation, lockdown and social isolation may have negative influences on the physical and mental well-being (Taylor, 2019) and experiencing anxiety and distress, isolation and lack of physical movement may also lead to fatigue. Against this background, the present study focuses on fatigue as one of the psychological components of a pandemic among the general population. More specifically, it aims to determine associations between knowledge, attitudes and practice concerning COVID-19 and psychological fatigue among the general population of Istanbul, the biggest city in Turkey, and to identify the risks and protective factors related to this psychological fatigue.

## Participants and methods

The study was conducted in the city of Istanbul, a large transcontinental municipality located in Eurasia, with a population approximating 16 million people. The study was a cross-sectional community-based survey among the population and was conducted in the hospitals, primary healthcare centres, family medicine clinics, universities and the governmental sector in the urban and semi-urban areas of Istanbul. This study was approved by the Clinical Research Ethics Committee of Istanbul Medipol University, Institutional Review Board (Research Protocol and IRB# 10840098-604.01.01-E.14180).

A multi-stage stratified random sampling method was performed from March to June 2020. A total of 4,700 persons were approached and 3,672 (78%) of participants (64.4% males and 35.6% females) completed the questionnaire, with ages ranging from under 30 to over 60 years.

The COVID-19 knowledge questionnaire was developed by the authors. The questionnaire is divided into five sections. The first part contains questions related to the socio-demographic details of the participants, the second part assesses the knowledge of the participants regarding COVID-19, the third part assesses attitude variables of the participants towards COVID-19 and the fourth part comprises questions related to precautionary measures taken by the participants in response to COVID-19. This part was designed on a true/false option basis where a *true* response was assigned 1 point, and a *false* response was assigned 0 points. The Cronbach’s alpha coefficient of the knowledge instrument was .85 in our study. The fifth part of the questionnaire is the Fatigue Assessment Scale (FAS), which is a simple 10-item self-reported questionnaire designed by Michielson et al. (2003) to assess general fatigue in the population and validated subsequently in the sarcoidosis setting. Five questions address physical fatigue and five questions mental fatigue (Drent et al., 2012; Michielsen et al., 2003). A total FAS score <22 indicates no fatigue (normal) and a score ⩾22 indicates fatigue. The internal consistency of the FAS was measured at .88 (Cronbach’s alpha coefficient), and none of the items would have improved the internal consistency if removed. In sum, FAS scores 10–21 = no fatigue (normal) and FAS scores 22–50 = fatigue.

Statistical test: data were analysed using the SPSS version 25 on percentages calculated for each categorical variable. Student’s *t* test was used to determine the significance of differences between the mean values of two continuous variables and confirmed by non-parametric Mann–Whitney test. The chi-square and Fisher’s exact tests (two-tailed) were performed to test for differences in proportions of categorical variables between two or more groups. Multivariate regression analysis was used to predict potential confounders and order the importance of risk factors (determinants) for COVID-19. All statistical tests were two-tailed and *p* < .05 was considered statistically significant.

## Results

Table 1 shows the demographic characteristics of subjects by fatigue and normal respondents. Of the total number of subjects surveyed, all recognized the term COVID-19 virus and claimed that they knew about the COVID-19 virus. There was a significant difference between fatigued and normal with respect to age, educational level, occupation status, place of residence as urban and rural, and number of family members (*p* < .001).

**Table 1.** Socio-demographic characteristics of fatigued and normal participants (N = 3,672).

Variables		Fatigued = 2,353	Normal = 1,319	*p* value significance
		*n* (%)	*n* (%)	
Age group	<30	553 (23.5)	280 (21.9)	
	30–39	680 (28.9)	460 (34.9)	
	40–49	660 (28.1)	370 (22.9)	<.001
	50–59	307 (13.0)	129 (10.1)	
	⩾60	153 (6.5)	70 (5.9)	
Gender	Male	1,516 (64.4)	817 (61.9)	.133
	Female	837 (35.6)	502 (38.1)	
Educational level				
	Primary	217 (9.2)	156 (6.8)	
	Preparatory	207 (8.8)	158 (7.7)	
	Secondary	687 (29.2)	338 (23.5)	.001
	University	972 (41.3)	526 (47.8)	
	Postgraduate MSc	183 (7.8)	102 (8.8)	
	Postgraduate PhD	87 (3.7)	39 (5.5)	
Occupation status				
	Sedentary	441 (18.7)	206 (15.6)	
	Businessman	167 (7.1)	84 (6.4)	
	Manual labour	232 (9.9)	164 (12.4)	
	Housewife	249 (10.6)	117 (8.9)	.001
	Professional	216 (9.2)	93 (7.1)	
	Police/military	102 (4.3)	51 (3.9)	
	Unskilled	131 (5.6)	85 (6.4)	
	Administrative/clerical	707 (30.0)	447 (33.9)	
	Retired/not working	108 (4.6)	72 (5.5)	
Monthly income				
	<3,000	901 (38.3)	516 (39.1)	
	3,000–4,999	780 (33.1)	451 (34.2)	.681
	>5,000–14,999	532 (22.6)	280 (21.2)	
	>15,000	140 (5.9)	72 (5.5)	
Place of residence				
	Urban	2,058 (87.5	1,223 (92.7)	.001
	Semi-urban	295 (12.5)	96 (7.3)	
Number of rooms				
	⩽3 rooms	1,468 (62.4)	831 (63.0)	.712
	>3 rooms	885 (37.6)	488 (37.0)	
Number of family members				
	⩽5 people	1,035 (44.0)	452 (34.3)	.001
	>5 people	1,318 (56.0)	867 (65.7)	

Table 2 indicates respondent’s knowledge of COVID-19 signs and symptoms. Responses indicated differences between fatigued (85.8%) and normal (89.5%) participants for knowledge of the clinical symptoms of COVID-19, including fever, dry cough and shortness of breath or difficulty breathing (*p* < .001). Most participants knew that there is no effective treatment/ vaccine for COVID-19, although early treatment may help most patients recover from the infection (89.8% of fatigued vs. 93.5% of normal; *p* < .001). More fatigued than normal participants believed that persons with COVID-19 cannot spread the virus to others when a fever is not present (35.5% of fatigued vs. 19.6% of normal; *p* < .001). Both groups believed that, to prevent infection by COVID-19, individuals should avoid going to crowded places (*p* = .008), and the isolation and treatment of people who are infected with COVID-19 are regarded as effective ways to reduce the spread of the virus (*p* = .002). People who have contact with someone who has contracted the COVID-19 virus should be immediately isolated in a proper place; meanwhile the observation period of isolation is considered to be 14 days (*p* = .002).

**Table 2.** Knowledge, attitude and practice towards COVID-19 by fatigued and normal participants (N = 3,672).

Knowledge of signs and symptoms	Fatigued = 2,353	Normal = 1,319	*p* value
	*n* (%)	*n* (%)	
1. The clinical symptoms of COVID-19 are fever, fatigue and dry cough	2,018 (85.8)	1,181 (89.5)	<.001
2. Do you agree that there is no effective treatment/vaccine for COVID-19	2,114 (89.8)	1,234 (93.5)	<.001
3. The COVID-19 infection can be contracted by contact with or eating wild animals	1,441 (61.2)	771 (58.5)	.098
4. The elderly, and people with chronic illnesses, diabetes, hypertension and obesity could be at risk	1,887 (80.2)	991 (75.0)	<.001
5. Do you think that persons with COVID-19 cannot transmit the virus to others when a fever is not present	788 (35.5)	259 (19.6)	<.001
6. Do you agree that the COVID-19 virus can spread via respiratory droplets of infected individuals	2,087 (88.7)	1,223 (92.7)	<.001
7. Wearing medical masks can prevent against the COVID-19 virus infection	2,091 (88.9)	1,179 (89.4)	.628
8. Should individuals avoid going to crowded places such as train, metro and bus stations to prevent infection by COVID-19	2,163 (91.9)	1,244 (94.3)	.008
9. The best treatment for those infected with the COVID-19 virus is isolation to reduce the spread, and the period of isolation considered is 14 days	2,172 (92.3)	1,252 (94.9)	.002
Attitude and behaviours			
1. Do you believe that COVID-19 will finally be successfully controlled	2,047 (87.0)	1,209 (91.7)	.001
2. Are you afraid to travel due to COVID-19	2,025 (86.1)	1,140 (86.4)	.756
3. Afraid of contact with people affected with COVID-19	1,980 (84.1)	1,112 (84.3)	.900
4. Do you feel that parents are responsible for teaching and guiding their children	2,104 (89.4)	1,225 (82.9)	.001
5. Are you satisfied with the preventive measures taken by the health authorities	1,993 (84.7)	1,193 (90.4)	<.001
6. Will you report to the medical authorities if a similar symptom related to COVID-19 is found in the community	2,102 (89.3)	1,235 (93.6)	<.001
7. Do you believe that the risk of COVID-19 is higher than for AIDS or cancer	1,724 (73.3)	859 (72.7)	.713
8. Do you trust that Turkey will overcome the pandemic COVID-19 virus	2,122 (90.2)	1,234 (93.6)	.001
Practice among people			
1. Recently, when you left home, did you wear medical masks and gloves to prevent against COVID-19 virus	2068(88.0)	11,213(92.0)	<.001
2. People should wash both their hands after coming from crowded places	2,144 (91.1)	1,262 (95.7)	<.001
3. Do you think that the eyes, nose and mouth can be affected with COVID-19 virus	2,196 (93.3)	1,262 (95.7)	.004
4. Are you taking advice from health professionals about COVID-19 virus	1,313 (55.8)	546 (41.4)	<.001
5. Do you think that Turkey needs extensive and frequent health education programmes on COVID-19 virus	1,969 (83.7)	1,072 (81.3)	.0.64
6 Do you avoid interacting with travellers coming from affected areas	2,084 (88.6)	1,273 (83.5)	<.001
7. Do you read and obey official public guidelines and announcements produced in your country about COVID-19	2,041 (86.7)	1,224 (92.8)	.001
8. People should strictly avoid going to crowded places	2,209 (93.9)	1,269 (96.2)	.002
9. Do you consider keeping physical distance to be isolation	2,139 (90.9)	1,246 (94.5)	.001
10. Do you believe in herbal medicine and treatment of COVID-19 with honey, lemon, mint, selenium, black seed oil, anise seeds, cinnamon and ground cloves	1,355 (57.6)	733 (55.6)	.237

Table 2 also reveals the attitude, behaviour and practices of subjects towards COVID-19 virus by fatigue. Nearly 85% of subjects were afraid of contacting people infected with COVID-19 and 86% were afraid to travel due to COVID-19 (fatigued 86.1% vs. normal 86.4%, *p* = .756). The majority of participants responded that risk of COVID-19 is higher than AIDS or cancer (73.3% of fatigue vs. 72.7% of normal; *p* = .713), and 87.0% of fatigued and 91.7% of normal believe that COVID-19 will finally be successfully controlled (*p* < .001). Most of the respondents (86.8%) were satisfied with the measures taken by the health authorities (84.7% of fatigued vs. 90.4% of normal; *p* <.001). Furthermore, they have confidence that Turkey will be able to overcome the pandemic of the COVID-19 virus (90.2% of fatigue vs. 93.6% of normal; *p* <.001).

Table 3 reveals the knowledge of appropriate methods for detecting COVID-19. The majority of participants consider the best methods of detecting COVID-19 to be antibody tests, computed tomography, sputum analysis and blood analysis, respectively.

**Table 3.** Knowledge of the appropriate methods for detecting COVID-19 by fatigue and normal participants (N = 3,672).

Appropriate methods for detecting COVID-19 virus	Fatigue = 2,353 *n* (%)	Normal = 1,319 *n* (%)	*p* value significance
1. Computed tomography	1,788 (76.0)	979 (74.2)	.234
2. Sputum analysis	1,792 (76.2)	993 (75.3)	.553
3. Blood analysis	1,752 (74.5)	960 (72.8)	.268
4. Urine analysis	552 (23.5)	311 (23.6)	.935
5. Physician diagnosis	1,583 (67.3)	895 (67.9)	.720
6. Antibody test	1,857 (78.9)	998 (75.7)	.023

Finally, Table 4 shows the multivariate stepwise regression analysis to determine the potential predictors of fatigue related to the COVID-19 pandemic. The analysis indicated that level of education, avoidance of going to crowded places (*p* < .001), believing that eye, nose and mouth are sensitive organs to the virus (*p* < .001), keeping physical distance is important for prevention (*p* < .001), isolation and treatment of infected people reduce the spread of COVID-19 (*p* < .003), COVID-19 is mainly transmitted through contact with the respiratory droplets of an infected person (*p* < .006), and the country should provide a health education programme for the COVID-19 virus. In addition, occupational status and the knowledge of antibody treatment variables were significantly associated with fatigue after adjusting for age, gender and income variables.

**Table 4.** Multivariate stepwise regression analysis to predict fatigue related to COVID-19 pandemic.

Independent variable	Regression coefficient	Standard error	*t*-test value	*p* value significance
Level of education	−0.030	0.007	−4.149	<.001
Avoid going to crowded places such as train, metro, and bus stations, and restaurants and shopping malls	−0.117	0.031	−3.729	<.001
Recognize COVID-19 disease and believe that the eyes, nose and mouth are organs sensitive to the virus	−0.090	0.026	−3.486	<.001
Keeping physical distance due to epidemic affect of COVID-19 virus	−0.118	0.030	−4.012	<.001
In recent days, have you gone to any crowded place and after returning washed hands and face with soap	–0.117	0.031	−3.729	.002
Isolation and treatment of people reduce the spread of virus	0.094	0.032	−3.977	.003
The COVID-19 virus spreads via respiratory droplets	−0.075	0.027	−2.764	.006
Occupation status	0.006	0.003	2.132	.033
The country should provide health education programmes	0.027	0.013	2.126	.035
Antibody treatment	0.040	0.019	2.076	.038

## Discussion

To the best of our knowledge, this is the first study in relation to fatigue and the COVID-19 pandemic conducted among the Turkish population. The study showed that 64.1% of the total participants are experiencing physical and mental fatigue, where fatigue was measured by the FAS questionnaire, which describes fatigue as feeling tired quickly, feeling mentally and physically exhausted, experiencing lack of energy, inability to start and perform everyday activities, lack of desire to do things, difficulty to think clearly and to concentrate on work (Satici et al., 2020).

The findings revealed that participants overall showed a correct rate of 87% of COVID-19 knowledge, indicating that most participants are well-informed about COVID-19. Generally, normal participants had more correct responses in terms of COVID-19 knowledge than fatigued participants. Between the fatigued and normal respondents, significant differences were observed in items related to knowledge of COVID-19: symptoms (fatigued 85.8% and normal 89.5%), believing there is no effective treatment (fatigued 89.8% vs. normal, 93.5%), virus can spread via droplets of spreading (fatigued 88.7% vs. normal 92.7%), transmission without fever is present (fatigued 35.5% vs. normal 19.6%), and prevention by avoiding crowded places (fatigued 91.9% vs. normal 94.3%) and isolation (fatigued 92.3% vs. normal 94.9).

Normal participants showed more positive attitudes than fatigued ones in terms of believing that COVID-19 will finally be controlled, satisfaction with preventive measures taken by the authorities, reporting suspected cases with symptoms and trusting that Turkey can overcome the COVID-19 pandemic. Normal participants also showed better practice in terms of wearing mask and gloves, washing hands, obeying guidelines and keeping physical distance. However, in terms of taking advice from health officials, avoiding interacting with travellers coming from affected areas was more common among the fatigued.

The COVID-19 pandemic has emerged unexpectedly and over 4 billion people are living in social isolation during this mother of all pandemics, and 6.7 million cases and about 400,000 deaths have been reported as of 6 June 2020, by WHO (2020a). In managing a pandemic, effective measures of quarantine, isolation and physical distancing are crucial intervention strategies. Recently, such measures have been reported as the most efficient ways to prevent the spread of the COVID-19 infection in Asia (Silva et al., 2020). Nevertheless, concerning mobility restrictions, a recent review by Brooks et al. (2020) reported the negative psychological effects of freedom constraints among those quarantined, such as post-traumatic stress symptoms, confusion and anger. Correspondingly, our results also suggest that practices such as avoidance of crowded places, washing hands, physical distancing and isolation are related to fatigue, as is knowledge related to means of transmission of the virus (i.e. virus spreads via respiratory droplets, and eyes, nose and mouth are sensitive organs). More recently, Satici et al. (2020) has found a significant positive relationship between fear of COVID-19 and depression, anxiety and stress. The present study also supports that experience of physical and psychological fatigue might be related to preventive restrictions of movement as well as fear and anxiety due to pandemic. Preventive measures, such as physical activity during periods of prolonged COVID-19 isolation and lockdown should be encouraged to support physical and mental health among the public (Matias et al., 2020).

The present findings also showed the level of education and occupational status as significant predictors of fatigue, indicating that people in some occupations might be more prone to experiencing fatigue and may need special preventive measures to enhance their physical and psychological well-being during the pandemic. Mental healthcare should be very comprehensive and should be accessible by a large population as it can influence the knowledge, attitude, behaviours, beliefs and practices in pandemic situations. The fear of infection and restrictions for prevention such as isolation can cause distress among the population and worsen those who have pre-existing psychiatric conditions (Taylor, 2019). Accordingly, particular preventive and supportive interventions for people with psychiatric conditions should be provided (Silva et al., 2020). The present study confirmed that the current global pandemic presents particular challenges to mental health among the public, thus, developing preventive strategies is urgently needed to enhance psychological resilience in the society (Silva et al., 2020). Unfortunately, during the COVID-19 pandemic, the number of people at risk is increasing dramatically, and, after the lockdown period ceases, mental health services are sadly expected to be overwhelmed day by day.

Furthermore, most of the guidelines are available to the public, healthcare providers, physicians, nurses, researchers and public health individuals by WHO (2020b) and the Centre for Disease Control (CDC, 2019; Silva et al., 2020; Sohrabi et al., 2020). Therefore, the public should strictly obey governmental rules and regulations regarding the preventive measures, managements and quarantine. More recently, a study from Hong Kong reported (Cheng et al., 2020) that wearing masks contributes to the control of COVID-19 by reducing virus shedding in saliva and respiratory droplets. This is consistent with the current survey where, when people leave home, the majority of the population (88% fatigued, 92% normal) wears surgical masks and gloves, and this can contribute to protection from the COVID-19 virus.

In the present global crisis situation caused by the COVID-19 pandemic, most individuals are exposed to unprecedented stressful conditions of unknown duration (Matias et al., 2020). This may not only increase daytime stress, anxiety and depression levels but also disrupt sleep. Managing sleep problems as best as possible during home confinement can limit stress and possibly prevent disruptions of social relationships (Altena et al., 2020) and fatigue.

The present study has several limitations. First, although it examined the associations between COVID-19 virus and KAP and many other factors related to health issues, the data were cross-sectional and therefore no conclusions can be made concerning issues of causality. Second, the KAP survey did not target the inclusion of specific types of participants, so it may not have provided the clearest responses or depiction of problems. Third, participants were not diagnosed by a psychiatrist. Finally, our sample was obviously over-representative of males. But the strengths of this study are that it addresses a very large sample encompassing a range of different sectors in society, it was conducted during a critical period of the COVID-19 outbreak and it is the very first research to investigate this topic in Turkey.

## Conclusion

The present epidemiological study has provided valuable information for understanding informative, attitudinal, behavioural, physical and psychological aspects of the COVID-19 pandemic in the case of the Turkish population. Experiencing fatigue can negatively affect a persons’ physical and psychological well-being and day-to-day activities and long-term fatigue might relate to psychiatric illnesses, particularly depression. The current study indicated that although knowledge, attitudes and behaviour concerning preventive measures, such as avoidance of crowded places and washing hands, are very important to prevent transmission of the disease, they are also associated with participants’ fatigue. Fatigue as a psychological outcome might be due to pandemic-related fear and anxiety. Policymakers and mental health professionals can inform the public on the possible fatigue experience as a psychological response during a pandemic, and suggest ways to cope and deal with physical and mental fatigue so as to enhance personal well-being during a pandemic situation.
